# Emerging Biomarkers for Screening and Management of Celiac Disease

**DOI:** 10.1155/2022/2756242

**Published:** 2022-05-28

**Authors:** Bilal Ahmad Mir, Tahir Majeed, Alka Singh, Mahendra Singh Rajput, Asheesh Kumar, Ashish Chauhan

**Affiliations:** ^1^Indira Gandhi Medical College, Shimla, India; ^2^All India Institute of Medical Sciences, New Delhi, India

## Abstract

Celiac disease (CeD) is a chronic, immune-mediated enteropathy that is precipitated by dietary gluten in genetically predisposed individuals expressing HLA-DQ2 and/or HLA-DQ8. In the current clinical practice, there are many serologic studies to aid in the diagnosis of CeD which include autoantibodies like IgA antitissue transglutaminase, antiendomysium, and antideamidated forms of gliadin peptide antibodies. Small intestinal biopsy has long been considered an essential step for the diagnosis of CeD. However, in the recent era, researchers have explored the possibility of CeD screening and diagnosis without endoscopy or biopsy. The newer emerging biomarkers of CeD appear promising in diagnostic evaluation and subsequent monitoring of disease. In this review of literature, we have explored the emerging biomarker-based diagnostic evaluation and monitoring of CeD.

## 1. Introduction

Celiac disease (CeD) is a chronic, immune-mediated multisystem disorder that is precipitated by gluten in diet of individuals with HLA DQ2 and/or DQ8 positivity [1–4]. The estimated global prevalence of CeD is very high (1–1.5%) [5]. CeD is characterized by villus atrophy of the duodenal mucosa associated with malabsorption of the nutrients and the subsequent clinical disease. The clinical spectrum of CeD includes intestinal as well as extraintestinal symptoms, such as anemia, fatigue, and dermatitis herpetiformis [6, 7]. In the past, typical CeD (now called classical CeD) denoted a clinical presentation with signs and symptoms of malabsorption, such as diarrhoea, steatorrhea, weight loss, and nutritional deficiencies. The term is of limited use as most of the patients do not present with so-called classical manifestations and a half may present with nondiarrhoeal form [8, 9]. Presentations previously described as atypical CeD and now termed nonclassical CeD (e.g., osteoporosis, anemia,, abdominal bloating, fatigue, and infertility) are now more common [7]. Asymptomatic CeD (also called silent CeD) is usually identified by using CeD-specific serology and is characterized by duodenal villous atrophy in individuals who lack symptoms or signs of CeD. Potential celiac disease denotes patients with normal small intestinal histology and positive CeD-specific serology [10, 11].

In the current clinical practice, there are many serologic studies to aid in the diagnosis of CeD which include autoantibodies like IgA antitissue transglutaminase (tTG), antiendomysial antibody (EMA), and antideamidated forms of gliadin peptide antibodies (DGP) [12]. Small intestinal histology showing villous atrophy has long been considered as an essential prerequisite for the diagnosis of CeD. The only proven treatment is adoption of a lifelong gluten-free diet (GFD), and clinical and histologic relapse occurs invariably when gluten is reintroduced. The genetically predisposing factors most extensively studied in CeD patients are HLA-DQ2 and/or HLA-DQ8, which are identified in almost 90-95% of patients [7].

## 2. A Case for Novel Biomarkers: Historical Prospective

Antigliadin antibodies (AGA) were the first serological marker for CeD that came into the picture in 1960s but soon went out of picture because of poor sensitivity and specificity. Both IgA and IgG antibodies were utilized in the diagnosis initially; however, presently, IgG AGA are in use for the diagnosis of nonceliac gluten sensitivity (NCGS). The remarkable step in celiac diagnosis came in the 1990s with the discovery of EMA that had a very high sensitivity and specificity of ~95%. But, EMA needed immunofluorescence which was cumbersome and required expertise that got solved with the invention of IgA anti-tTG with almost similar diagnostic accuracy. Now, we have IgA anti-tTG, antideamidated gliadin peptide (anti-DGP), and EMA that are most commonly used in the present scenario for diagnosis. In the last two decades, we have progressed from serology-based tests to point-of-care testing (POCT), genetic testing, testing of antibodies in other fluids (saliva, faeces, and stools), and tests to ascertain villous atrophy (I-FABP and citrulline).

## 3. Novel Biomarkers

### 3.1. Point-of-Care Testing

Point-of-care testing (POCT) has revolutionized the diagnosis of various diseases. Card-based POCTs have made diagnosis of CeD rapid and easy. A study by Lau et al. explored the role of Sintex POCT, based on detection of DGP either IgA/IgG via immunochromatographic principle. It is a card-based test with three lines in the card: one detecting the presence of IgA/IgG against DGP, another detecting serum IgA, and a control line. This test requires 25 mcg/L of blood by finger prick and showed a sensitivity and specificity comparable to IgA tTG and EMA in symptomatic patients. Moreover, patient preference was markedly in favour of POCT vis a vis serological tests (90% vs. 2.8%) [13]. In another study from India involving the pediatric population, Biocard, a lateral flow immunochromatographic strip system, showed a sensitivity and specificity of 83% and 93%, respectively, against a gold standard of combination of duodenal biopsy and EMA [14]. In a meta-analysis by the same group, the sensitivity and specificity of tTG/DGP/tTG+AGA-based tests was 90% and 95%, respectively [15]. Hence, it could be concluded that in resource constrained settings, POCTs are reliable methods to diagnose CeD.

### 3.2. Detection of CeD Autoantibodies in Saliva and Faeces

Saliva and stool samples are an excellent specimen for screening of CeD, as these samples are easily obtained by noninvasive methods and do not need a venepuncture. A test available to measure IgA anti-tTG in saliva consists of a fluid-phase radioimmunoassay method. It has been shown to have a good sensitivity and specificity in a study involving 5000 children, where 31 out of 32 serology-positive children had positive salivary assay [16]. Despite a good diagnostic accuracy, the test has inherent problems associated with the use of radioisotopes as well as radioactive waste disposal. Adornetto et al. described an enzyme-linked immunomagnetic electrochemical assay for measuring IgA anti-tTG in saliva, based on magnetic beads to support the immunological chain reaction and differential pulse voltammetry as the detection technique. This method has high specificity and sensitivity, bypassing the problems intrinsic to the radioimmunoassay method [17]. Although these results are encouraging, these tests need more data to recommend their use as a method of screening.

Studies have explored stools as a possible sample for detecting CeD antibodies. Di Tola et al. showed that the area under the curve (AUC) for IgA anti-tTG (AUC = 0.862, *p* < 0.0001), IgA anti-DGP (AUC = 0.822, *p* < 0.0001), and IgA/IgG tTG/DGP (AUC = 0.783, *p* = 0.0003) in faecal samples are very significant [18]. However, the sensitivity of 76% for faecal IgA antibodies against tTG makes it unsuitable as a screening for CeD.

### 3.3. Intestinal-Fatty Acid Binding Protein (I-FABP)

I-FABP is a small cytosolic protein (15 kDa) and serves as a marker for enterocyte damage. It is present in mature enterocytes and on enterocyte damage gets rapidly released into the circulation. I-FABP is most commonly found in the small intestine, jejunum in particular and that too at the distal villi that are the site of early damage in CeD. Therefore, circulating I-FABP is a surrogate marker for the extent of intestinal epithelial cell injury. It is a valuable marker in the evaluation of intestinal epithelial damage in various disease states such as mesenteric infarction, intestinal ischemia, and necrotising enterocolitis [19, 20]. Studies have shown that patients with untreated CeD have elevated levels of I-FABP, and these levels normalize after initiation of a GFD [21–23]. In a study involving patients with up to 10-fold tTG rise and villous atrophy vis a vis patients without villous atrophy and only tTG rise, mean I-FABP levels were significantly higher in patients with villous atrophy (784.7 pg/ml vs. 172.7 pg/mL, *p* < 0.001) and I-FABP levels declined on GFD [21]. Moreover, I-FABP levels recovered rapidly on GFD, implying that plasma I-FABP may also be used for monitoring disease activity in CeD patients on a GFD. The positive predictive value for CeD of an increased I-FABP level in children with elevated CeD autoantibody titres and HLA-DQ2 and/or -DQ8 positivity was 100%. The negative predictive value of I-FABP in this group was 50% with a sensitivity and specificity of 84.7% and 100%, respectively, for the detection of CeD in these patients [23]. In another study involving 68 children (CeD = 49 and controls = 19) with raised IgA tTG, I-FABP concentration was significantly higher in cases than controls (458 pg/ml vs. 20 pg/ml), only 2 out of 19 controls had raised I-FABP, and out of them one later turned out to be celiac and I-FABP correlated with the degree of villous atrophy [22]. In a study from India, diagnostic accuracy of I-FABP >1100 pg/ml was 78% in a cohort of celiac patients, and this value decreased on GFD [24]. Hence, I-FABP has been proposed as a marker for no biopsy approach in patients not qualifying for 10-fold tTG rise [22].

### 3.4. Plasma Citrulline

Citrulline, a nonessential amino acid, is synthesized specifically in small intestinal enterocytes; hence, its levels are representative of the synthetic function of enterocytes. The first evidence of the role of citrulline in assessing enterocyte mass was shown by Crenn et al. in patients of short bowel syndrome who had significantly raised levels in comparison to controls (20 ± 13 vs. 40 ± 10 *μ*mol L, *p* < 0.001) and levels of citrulline also correlated with the length of the resected intestine [25]. Evaluating the role of citrulline in disorders other than short bowel, Crenn et al. reported its value in correlation to villous atrophy. Values of <10 *μ*mol/L, 10-20 *μ*mol/L, and 20-30 *μ*mol/L correlated with total villous atrophy, proximal only villous atrophy, and partial villous atrophy, respectively [25]. In a meta-analysis by Fragkos and Forbes, plasma citrulline level of 20 *μ*mol/L had a sensitivity and specificity of ~80%. But, in inflammatory states, citrulline may be decreased without any intestinal malabsorption as nitric oxide and arginine gets depleted in inflammatory states leading to reduction in citrulline [26]. In a recent study from India, plasma citrulline level < 30 *μ*mol/L had a diagnostic accuracy of 89% with a sensitivity and specificity of 95% and 90%, respectively, for predicting villous atrophy of Marsh grade > 2 [24]. Hence, citrulline may be used as a marker to predict villous atrophy in patients unwilling for biopsy and for follow-up of patients on GFD.

### 3.5. HLA Typing (Genetic Screening)

The cohort of patients with CeD who do not have classical symptoms of CeD gave birth to an idea called “celiac iceberg.” Experts have proposed expansion of the “iceberg” to include patients who are genetically susceptible to CeD, i.e., HLA DQ2- and/or HLA DQ8-positive patients [27, 28]. ESPGHAN-2012 had proposed “triple test” strategy for no-biopsy approach of diagnosing CeD, i.e., very high fold IgA-tTG serology, EMA-IgA positivity, and HLA DQ2/DQ8 positive [3]. The recent ESPGHAN-2020 guidelines have removed the HLA typing from the no-biopsy approach of diagnosing CeD [29]. The basis of this stems from multiple European studies that have shown no additional benefit of doing HLA typing over and above high tTG and EMA [30, 31].

Genetic screening provides a novel methodology that could be used to obtain accurate estimates of the at-risk individuals of CeD. The screening also detects false-positive IgA tTG serology in adults at average risk of CeD as CeD is almost always found in patients possessing genes encoding either HLA-DQ2.5, DQ2, or DQ8. HLA typing has a high negative predictive value for CeD albeit a very poor positive predictivity as 30%-40% of the general population harbours these genes [32]. Up to 90%-95% of patients with CeD in Western cohorts have HLA-DQ2 heterodimer (HLA-DQ2.5), encoded by DQ A1∗0501 and DQB1∗0201 alleles. HLA DQ8 accounts for the remaining 5%-10% [33]. In a Western cohort, only 0.5% of CeD patients were HLA DQ2/DQ8 negative, emphasizing that populations without DQ2/DQ8 genes have a very less chance of developing CeD [34]. Hence, genetic testing is an important tool in excluding CeD in cases of diagnostic dilemma.

### 3.6. Neoepitopes of Tissue Transglutaminase and Deamidated Gliadin Peptide

A recent study has shown a complex of tTG- and DGP-synthesized peptides to be a method of high diagnostic accuracy for CeD, with a sensitivity and specificity found to be 99% and 100%. These neoepitopes showed more reactivity in patients on GFD with healed mucosa in comparison to patients with unhealed mucosa. The overall diagnostic accuracy of these epitopes in diagnosing villous atrophy in patients on GFD was 90% which was better than other serological tests [35]. Though promising, this biomarker needs further data and validation in different racial cohorts for its use for screening/monitoring response in preference to antibodies that are presently in use.

### 3.7. HLA-DQ-Gluten Tetramers

A lot of people now are or on self-prescribed GFD, and diagnosing CeD in this subset is a problem as serology and histology has less accuracy in these patients. Guidelines recommend a gluten challenge with 3-6 grams of gluten in these patients prior to any serology testing or duodenal biopsy. Detection of HLA-DQ-gluten tetramers in blood detects CD4-positive T-cell reactivity in patients of CeD and has been found to have good diagnostic accuracy in patients already on GFD. These tetramers have a high sensitivity and specificity for patients on GFD, i.e., 100% and 90%, respectively. Hence, for patients on a gluten-containing diet, this test offers comparable sensitivity and specificity via antibody tests [36].

### 3.8. Peptide-Functionalized Gold Nanoparticles

Gold nanoparticles (AuNPs) are small gold particles with a diameter of 1 to 100 nm. There are sensing platforms based on the optical characteristics of AuNPs for the molecular detection and recognition of disease biomarker. Colorimetric sensors based on AuNPs have been applied for identifying targets, DNA, protein conformations, and enzyme activity, where they have demonstrated high sensitivity and effectiveness. There has been advent of newer designs of these nanoparticles that have more enhanced and controlled surface chemistry to be used for sensing applications. Peptide-functionalized nanoparticles (PFNs) are one of the prototype of such sensing platform [37].

Recent studies have demonstrated the potential of PFNs as a colorimetric sensor, using AuNPs coated with a peptide sequence derived from the gliadin protein, for screening CeD. A deamidated peptide sequence is derived from *α*-gliadin amino acids that detects the immunogenic peptide sequence acting as a trigger for CeD [37]. The AuNP peptide assay seems promising for development as a POCT as it is based on the formation of a precipitate, and there occurs a reduction in color of a positive sample in the presence of a CeD-specific autoantibody, with an overall accuracy of 86.6% [38].

### 3.9. Regenerating Gene Ia

The regenerating gene (REG) is a multigene family in humans and encodes small multifunctional secretory proteins that might be involved in cell proliferation, differentiation, and regeneration. The REG Ia protein is expressed in the liver, pancreas, and the gastrointestinal tract [39]. Recent data has shown that REG Ia levels are significantly higher in sera of CeD patients, while its levels are not increased in other autoimmune diseases like pernicious anemia and T1DM, and serum REG Ia levels decrease on GFD. Microarray analysis demonstrates that GFD in CeD patients reverses the altered transcription of genes in small-bowel biopsy samples, suggesting that the detected alterations in CeD are caused by the reaction to gluten and not by a primary defect [40]. The decrease in REG Ia levels in sera after GFD coincides with the fall of autoantibodies to transglutaminase. The decrease of REG Ia levels in sera correlates well with the decrease of IgA anti-tTG levels as both start to decline soon after removal of gluten [41]. REG Ia appears to be a promising biomarker for CeD that can help in both diagnosis and to ascertain compliance with the GFD.

### 3.10. CD3 Immunohistochemical Stain

The use of CD3 IHC expression represents a sensitive and specific tool to distinguish IELs from epithelial cells especially in Marsh 1 cases because the occurrence of IELs by itself is not specific for CeD and can be observed in other forms of intestinal inflammations. Current studies show that ≥30 IEL/100 epithelial cells are detected in 68 to 100% of CD3 positive cases, i.e., CD3+ [42].

There is a significant relationship between the count of CD3+ T-lymphocytes per 100 epithelial cells and the histopathological changes in the duodenal biopsy according to Marsh classification. Moreover, the immunohistochemical staining of CD3 in intraepithelial T-lymphocytes could help in definite assessment in 43.3% of the patients with Marsh grade 1 histological lesion. In addition to that, the IHC expression of CD3+ marker provides a hint about its distribution of within the lymphocyte whether global surface or clonal surface and intracytoplasmic to diagnose refractory CeD [42].

### 3.11. Mucosal IgA-tTG and EMA Deposits

tTG antibodies are produced primarily at an intestinal level by specific B lymphocytes, and once produced, tTG antibodies are deposited in the small intestinal mucosa, even before they can be detected in the bloodstream [12]. Therefore, these autoantibody deposits in biopsies can ascertain the diagnosis in borderline cases, primarily in patients with seronegative CeD [43]. In this setting, also the EMA assay in cultured intestinal mucosa biopsies before and after an in vitro gliadin challenge may be an additional tool to either confirm or exclude the presence of a gluten-related enteropathy [44]. It is important to note that IgA-tTG is produced in small intestine, and hence, the deposits preclude the development of a positive IgA-tTG serology [12]. These deposits are helpful in predicting progression of potential CeD to CeD and may help in a case where diagnosis cannot be made on biopsy and serology. Flow cytometry of intestinal epithelial lymphocytes showed increased IELs in active CeD and a 97% specificity for CeD diagnosis [45]. We had shown the utility of these deposits in establishing these deposits in the esophagus, stomach, and colon and found significantly more deposits at these sites in comparison to the controls [46]. We have also shown the utility of these deposits in patients with celiac-related liver disease.

## 4. Conclusion

Prior to the advent of serology, diagnosis of CeD was cumbersome and required biopsy in all cases. The need to avoid biopsy was a great impetus for the scientific community to look for novel biomarkers. Nowadays, the increased diagnostic accuracy of the newly emerged plasma biomarkers and those in the pipeline suggests a paradigm change in adult CeD diagnosis. There is, however, the need for more data to predict villous atrophy and obviate the need of biopsy. We also need biomarkers for diagnosing CeD with good accuracy in special subgroups of patients such as those with seronegative CeD, patients already on GFD, and borderline patients for the diagnosis of CeD. We also need more biomarkers for predicting villous atrophy that obviates the need for biopsy as well as for monitoring of the disease.
